# Skeletal Muscle Membrane Permeability Markers Derived From ^31^P‐MRS May Reflect Disease Activity in Becker Muscular Dystrophy

**DOI:** 10.1002/nbm.70155

**Published:** 2025-10-07

**Authors:** Esther J. Schrama, Melissa T. Hooijmans, Nienke M. van de Velde, Erik H. Niks, Hermien E. Kan, Donnie Cameron

**Affiliations:** ^1^ Department of Neurology Leiden University Medical Center Leiden the Netherlands; ^2^ Duchenne Center Netherlands; ^3^ Department of Human Movement Sciences, Faculty of Behavioural and Movement Sciences VU University Amsterdam the Netherlands; ^4^ C.J. Gorter MRI Center, Department of Radiology Leiden University Medical Center Leiden the Netherlands; ^5^ Department of Medical Imaging Radboud University Center Nijmegen the Netherlands

**Keywords:** ^31^P‐MRS, Becker muscular dystrophy, cell membrane permeability, diffusion‐tensor MRI, magnesium, skeletal muscle

## Abstract

Replacement of muscle tissue by fat in patients with Becker muscular dystrophy (BMD), as measured by quantitative muscle MRI, has been shown to reflect disease progression, but this process is considered irreversible. To monitor treatment effects in healthy‐appearing muscles, biomarkers reflecting disease activity are needed. Here, we compare several candidate biomarkers for disease activity between patients with BMD and controls: intracellular ionised magnesium ([Mg^2+^]), phosphodiesters (PDE), and weighted pH measures from phosphorus‐(^31^P)‐MRS; and membrane permeability derived from the random permeable barrier model (RBPM), as applied to diffusion‐tensor‐(DT)‐MRI data. We performed 7 T ^31^P‐MRS and 3 T DT‐MRI in the left lower leg of 23 participants with BMD (mean [range] age: 41.1 [18.8–66.2] years) and 14 healthy controls (mean [range] age: 43.0 [21.2–63.6] years), estimating [Mg^2+^], PDE/γ‐ATP, weighted pH and RPBM permeability. Follow‐up scans at 24 months were performed in a subset of these participants. Muscles in patients with BMD were regarded as likely to be ‘preserved’ (BMD_pre_) with fat fractions ≤ 13.5% and as ‘progressing’ (BMD_prog_) with 13.5% < fat fraction < 81.5%. Muscles with fat fractions ≥ 81.5% were excluded from further analyses. We observed decreased [Mg^2+^] in BMD_pre_ and BMD_prog_ compared to healthy controls, whereas PDE/γ‐ATP and weighted pH were increased in these muscles. RBPM‐measured permeability did not differ between groups. We observed no longitudinal changes in [Mg^2+^], PDE/γ‐ATP or weighted pH. Only [Mg^2+^] continued to show group differences on inclusion of longitudinal data, and weighted pH demonstrated inter‐muscle differences. In patients with BMD, ^31^P‐MRS demonstrates reduced [Mg^2+^] and increased weighted pH in the lower leg muscles versus controls, suggesting greater membrane permeability—a potential disease activity biomarker independent of the disease phase. PDE/γ‐ATP was also significantly increased in progressing and preserved muscle. Incorporating ^31^P‐MRS in therapeutic trials will help to further establish its use as a response biomarker.

## Graphical abstract

Biomarkers for muscle disease activity are needed for trials in Becker muscular dystrophy (BMD). We investigated magnesium (Mg^2+^), phosphodiesters (PDE) and pH from ^31^P‐MRS; and membrane permeability from random permeable barrier model (RBPM) diffusion as candidates, studying ‘preserved’ and ‘progressing’ muscles in patients with BMD versus controls.

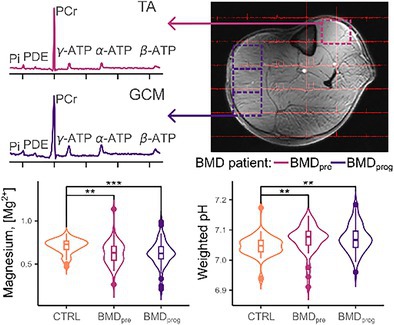

**Abbreviations**
^31^P‐MRS: phosphorus magnetic resonance spectroscopy
ANOVA: analysis of variance
ATP: adenosine triphosphate
BMD: Becker muscular dystrophy
BMD_end_
: end‐stage muscles in BMD
BMD_pre_
: preserved muscles in BMD
BMD_prog_
: progressing muscles in BMD
CSI: chemical shift imaging
CTRL: healthy control
DMD: Duchenne muscular dystrophy
DT‐MRI: diffusion‐tensor‐magnetic resonance imaging
EMA: European Medicines Agency
FDA: U.S. Food and Drug Administration
GCL: gastrocnemius lateralis
GCM: gastrocnemius medialis
[Mg^2+^]: free ionised magnesium concentration
PCr: phosphocreatine
PDE: phosphodiesters
PER: peroneus muscle
Pi: inorganic phosphate
RPBM: random permeable barrier model
SE: spin echo
SNR: signal‐to‐noise ratio
STE: stimulated echo
SOL: soleus
TA: tibialis anterior
TP: tibialis posterior

## 1 Introduction

Becker muscular dystrophy (BMD) is a rare X‐linked neuromuscular disorder caused by pathogenic variants in the dystrophin (*DMD*) gene. BMD is characterised by progressive muscle wasting and weakness, where muscle fibres are more susceptible to damage and are eventually replaced by fat and fibrotic tissue. Disease progression is slow and highly variable, making therapeutic trials difficult to power in order to capture meaningful changes in clinical outcome measures. In Duchenne muscular dystrophy (DMD), a more severe form of dystrophinopathy, skeletal muscle dystrophin is accepted by the U.S. Food and Drug Administration (FDA) as a surrogate endpoint for antisense oligonucleotide trials [1]. For BMD, no surrogate endpoints have been accepted to date, but their need has been recognised. The European Medicines Agency (EMA) defines biomarkers as “measurable characteristics that are indicators of normal biologic processes, pathogenic processes, and/or response to therapeutic or other intervention” [2]. Fat fraction, as measured by chemical‐shift‐based water‐fat‐separation MRI, reflects disease progression and has been proposed as a strong candidate biomarker for determining prognosis and assessing treatment efficacy [3]. However, replacement of muscle tissue by fat is considered an irreversible end stage of muscle pathology. Ideally, interventions should be administered prior to loss of muscle function to preserve as much muscle tissue as possible. This requires biomarkers that focus on *disease activity*: a term that includes processes such as muscle inflammation, necrosis and regeneration [4]. Membrane leakiness is one such process that has been relatively underexplored to date, despite being accessible via in vivo MRI measurements.

A number of MRI methods have been employed for non‐invasive assessment of muscle membrane permeability, which is linked to membrane leakiness. Early work in preclinical models of muscular dystrophy used albumin‐targeted gadolinium to monitor sarcolemmal integrity [5, 6, 7]; however, this approach was never translated to human studies. In DMD, ionic homeostasis is known to be disturbed [8], leading to reduced intracellular Mg^2+^ as measured by phosphorus‐(^31^P)‐MRS [9]. Free ionised magnesium therefore merits assessment as a potential biomarker for muscle membrane permeability [9, 10], alongside other ^31^P‐MRS‐derived metrics, such as phosphodiesters (PDE), which reflect membrane phospholipid breakdown, and weighted pH. Diffusion MRI has also seen numerous applications to skeletal muscle, and offers direct estimation of permeability in physical units, from Tanner's early work in frog muscle [11] to applications of the random permeable barrier model (RPBM) [12] in compartment syndrome, training, muscle atrophy and BMD [13, 14, 15, 16]. The RBPM can also assess fibre diameter using multi‐diffusion‐time diffusion‐tensor‐(DT)‐MRI data.

Our group has previously explored multiparametric MR as an early disease marker for BMD, and found PDE measured by ^31^P‐MRS was increased in some muscles compared to controls [17, 18]. Other metabolic indices and DT‐MRI revealed no significant changes, but [Mg^2+^] and the RBPM were not analysed. Our aim in this study was to reanalyse our ^31^P‐MRS and DT‐MRI data to explore [Mg^2+^] and the RPBM, both of which reflect membrane permeability, as possible biomarkers for disease activity. We compared intramuscular [Mg^2+^], weighted pH and PDE/γ‐adenosine‐triphosphate (ATP) ratios measured using ^31^P‐MRS, and RPBM membrane permeability measured using DT‐MRI in the lower leg between relatively preserved muscles and progressing muscles in BMD and healthy controls. Further, we assessed changes in the ^31^P‐MRS parameters at 24‐month follow‐up.

## 2 Methods

### 2.1 Participant Cohort

We included all participants who underwent 7 T ^31^P‐MRS in our previously published MRI study in BMD [18]: namely, 23 patients with BMD and 13 healthy controls. In short, patients with BMD were recruited from the Dutch Dystrophinopathy Database [19]; the inclusion criteria were as follows: Male sex and genetically confirmed BMD diagnosis based on pathogenic variants in the *DMD* gene, either in‐frame or other with a mild phenotype (ambulant beyond 16 years of age). Healthy male controls, recruited from a local Department of Radiology database, were excluded if their medical history contained a neuromuscular disorder. Out of all participants, 24‐month follow‐up data were available for 17 patients with BMD and seven controls.

In addition to ^31^P‐MRS data, we also included 3 T multi‐diffusion‐time DT‐MRI datasets that were acquired at 24‐month follow‐up in a subset of participants: 13 patients with BMD and nine controls.

Further, chemical‐shift‐based water‐fat‐separation or ‘Dixon’ acquisitions were applied at 3 T in all participants to determine fat fractions of all lower leg muscles.

Ethical approval was granted by the local research ethics committee (protocols P10.133, P12.214, and P14.243), and participants gave written informed consent after receiving a detailed description of the study, in accordance with the Declaration of Helsinki.

### 2.2 Magnetic Resonance Imaging

#### 2.2.1 Phosphorus‐31 Magnetic Resonance Spectroscopy

Phosphorus MRS experiments were performed, as described before, on a 7 T MRI scanner (Achieva, Philips Healthcare, Best, The Netherlands) using a dual‐tuned ^1^H/^31^P transmit‐receive volume coil [20]. Participants were set up in supine, feet‐first orientation. Data were obtained in the left lower‐leg and a copper sheet was wrapped around the contralateral leg to minimise spectral contamination.

After localisers, T_1_‐weighted anatomical acquisitions were applied as a reference for chemical shift imaging (CSI) and for identifying muscle groups in the lower leg. Imaging parameters were as follows: 2D spoiled gradient‐recalled echo with TR = 10 ms, TE = 3.1 ms, flip angle = 30°, field‐of‐view = 203 mm × 203 mm, matrix size = 224 × 210, 15 slices with 8 mm slice thickness and reconstructed in‐plane resolution = 0.47 mm × 0.47 mm.

Anatomical imaging was followed by second‐order shimming using a localised shimming tool [21]. The optimal ^31^P transmit gain required to obtain a 90° RF pulse was determined using a series of pulse‐acquire experiments with a linearly increasing flip‐angle. A ^31^P 2D CSI acquisition was then played, with the following settings: TR/TE = 2000/0.5 ms, field‐of‐view = 160 × 200 mm, matrix size = 8 × 10, 2048 complex data points, bandwidth = 4000 Hz, block excitation with FA = 45° and accumulation‐weighted phase‐encoding [22] with 24 NSA at the centre of *k*‐space. The acquisition used a non‐selective excitation, and thus included the whole sensitive volume of the birdcage coil, which was 12 cm in length.

#### 2.2.2 Diffusion‐Tensor and Water‐Fat‐Separation Imaging

Diffusion and water‐fat imaging experiments were performed using a wide‐bore 3 T MRI scanner (Ingenia, Philips Healthcare, Best, The Netherlands) with a maximum gradient amplitude of 45 mT/m and a gradient slew rate of 200 T/m/s. Images were acquired using the body coil for transmission and a combination of a flexible 16‐element anterior torso array and a 12‐element built‐in posterior array for reception. Participants were again set up in feet‐first supine orientation with their ankles supported by sandbags. For assessment of skeletal muscle fat replacement, a chemical‐shift‐based water‐fat‐separation, or ‘Dixon’, acquisition was performed in the left lower leg, centred on the thickest part of the calf. Scan parameters were as follows: 2D spoiled gradient‐recalled echo sequence, TR = 210 ms; 3 echoes, with TE_1_ = 4.4 ms and ∆TE = 0.8 ms; flip angle = 8°; field‐of‐view = 180 mm × 180 mm; matrix size = 180 × 180; reconstructed in‐plane resolution = 0.47 mm × 0.47 mm; 23 slices with 10 mm thickness and a 5 mm gap; and 2 signal averages. This was followed by spin‐echo‐(SE)‐ and stimulated‐echo‐(STE)‐DT‐MRI scans with: TR/TE = 5000/58 ms; field‐of‐view = 384 × 384 mm; matrix size = 96 × 96; 9 slices, 6 mm thickness, 3 mm gap; *b*‐values = 0 and 400 s/mm^2^; 12 diffusion directions; SENSE factor = 1.7; diffusion times, Δ = 27, 130, 330 ms; and comprehensive fat suppression [23].

### 2.3 Phosphorus‐31 MRS Data Processing

The ^31^P 2D CSI data were first filtered using a Hann window in MATLAB (version 2021b, The Mathworks, Natick, USA) to minimise signal contamination from adjacent voxels at the expense of a larger true voxel size.

#### 2.3.1 CSI Voxel Selection

The filtered 2D‐CSI data were loaded in the 3D Interactive Chemical Shift Imaging tool (3DiCSI, version 1.911, Columbia University, NY, USA), together with the anatomical T_1_‐weighted images, for selection of muscle‐specific spectra. Voxels were selected for larger muscles in the lower leg: the tibialis anterior and posterior (TA and TP), peroneus (PER), soleus (SOL) and gastrocnemius lateralis and medialis (GCL and GCM). For each muscle, the CSI grid was shifted by half‐voxel increments to position voxels fully within the muscle, both in‐plane and in the slice direction. The extensor digitorum longus was not large enough to merit a single voxel without introducing significant partial volume from other muscles. For the largest muscles—the soleus, GCL, and GCM—up to four voxels were selected for averaging; in these cases, the CSI grid was not repositioned during voxel selection, to ensure voxels did not overlap. For each voxel, spectra were individually zero‐ and first‐order phase corrected before being exported in text format for further processing. Voxel selection in follow‐up data was matched to baseline voxel selection with the aid of screenshots.

#### 2.3.2 Spectral Quantification

Automatic spectral preprocessing was performed in Python (version 3.10, Python Software Foundation, www.python.org) and was based on a previous pipeline [24] with the addition of functions from the FSL‐MRS (git.fmrib.ox.ac.uk/fsl/fsl_mrs) [25] and nmrglue (www.nmrglue.com) [26] packages. The code used in this manuscript is available at https://git.lumc.nl/neuroscience/p31mrs_magnesium. Briefly, signal‐to‐noise ratios (SNRs) were conservatively estimated using the height of the phosphocreatine (PCr) peak in the frequency domain and the standard deviation of the noise in the time domain. Spectra with an SNR < 10 were excluded from further processing. The retained spectra were zero‐ and first‐order phase‐corrected using the ACME algorithm [27], as implemented in nmrglue's proc_autophase module. When automatic phase correction failed, manual phase correction was performed interactively using the manual_ps function in nmrglue. Spectra were then apodised with a 20 Hz Lorentzian filter, two‐times zero‐filled, and the PCr peak was set to 0 ppm. Where multiple spectra were available for a single muscle, these spectra were frequency‐ and phase‐aligned using the phase_freq_align function in FSL‐MRS prior to averaging. Two sets of intermediary reports and plots were produced for quality control: a report to evaluate frequency and phase alignment (phase_freq_align_report), and a plot of the processed spectrum, to permit assessment of spectral quality. After preprocessing, the AMARES time‐domain‐fitting algorithm from jMRUI (version 3.0) was run within the pipeline, in batch mode, to determine the amplitudes of the PCr; γ‐, α‐ and β‐ATP; phosphodiesters (PDE); and inorganic phosphate (Pi) resonances in the time domain. To account for the spectral baseline during fitting, the first 12 points of the signal were multiplied by a quarter sine wave. Metabolite ratios, weighted pH^10^ and [Mg^2+^] were then calculated.

The chemical shifts of inorganic phosphate resonances Pi_a_ and Pi_b_ were used to calculate pH values via the modified Henderson–Hasselbalch equation:

(1) $$\mathrm{pH}=6.75+\log\frac{3.27-\sigma_{1}}{\sigma_{1}-5.69}$$

where *σ*_1_ is the chemical shift difference of the Pi peaks relative to phosphocreatine. The weighted pH was then calculated as described by Reyngoudt et al. [10], using pH values determined for Pi_a_ and Pi_b_:

(2) $$pH_{\mathrm{wt}}=pH_{a}\cdot \frac{Pi_{a}}{Pi_{\mathrm{tot}}}+pH_{b}\cdot \frac{Pi_{b}}{Pi_{\mathrm{tot}}}$$

Free ionised intracellular Mg in muscle, [Mg^2+^] in millimolar units, was estimated using the Newton function in SciPy to determine the roots of the following quadratic equation [28]:

(3) $$\begin{array}{c} \begin{array}{c} \delta_{\mathrm{obs}}^{\alpha -\beta }=\frac{\delta_{\mathrm{ATP}}^{\alpha -\beta }+\delta_{\text{HATP}}^{\alpha -\beta }\cdot K_{H}\cdot \left[H^{+}\right]+\delta_{\text{MgATP}}^{\alpha -\beta }\cdot K_{\mathrm{Mg}}\cdot \left[Mg^{2+}\right]+\delta_{\text{MgHATP}}^{\alpha -\beta }\cdot K_{H}\cdot K_{\mathrm{MgH}}\cdot \left[H^{+}\right]\left[Mg^{2+}\right]+\delta_{Mg_{2}\mathrm{ATP}}^{\alpha -\beta }\cdot K_{Mg_{2}}\cdot K_{\mathrm{Mg}}\cdot {\left[Mg^{2+}\right]}^{2}}{1+K_{H}\cdot \left[H^{+}\right]+K_{\mathrm{Mg}}\cdot \left[Mg^{2+}\right]+K_{H}\cdot K_{\mathrm{MgH}}\cdot \left[H^{+}\right]\left[Mg^{2+}\right]+K_{Mg_{2}}\cdot K_{\mathrm{Mg}}\cdot {\left[Mg^{2+}\right]}^{2}} \end{array} \end{array}$$

where $\delta_{\mathrm{obs}}^{\alpha -\beta }$ is the observed chemical shift of β‐ATP relative to α‐ATP, $\left[H^{+}\right]=10^{-pH_{\mathrm{wt}}}$, and formation constants K and chemical shifts δ are taken from the literature [29].

The ratio of PDE and γ‐ATP was calculated as a marker of cell membrane damage. Glycerol 3‐phosphocholine and glycerol 3‐phosphoethanolamine both contribute to the PDE signal and are thought to reflect membrane phospholipid breakdown [30].

Finally, metabolite ratios were corrected for T_1_ saturation, using the 7 T ^31^P metabolite T_1_ values determined by Bogner et al. [31].

After post‐processing, quality‐control reports and plots were checked manually for issues and artefacts such as peak‐splitting, ‘feet’ [32], and missing resonances by a clinician researcher with 3 years of experience in MRI (E.J.S.). Where quality issues were deemed to affect the measured [Mg^2+^] and metabolite ratios, spectra were excluded from further analysis. Borderline cases were discussed with an expert with over 14 years' experience in ^31^P‐MRS data analysis (D.C. or H.E.K.).

### 2.4 DT‐MRI Data Processing

DT‐MRI data were processed in MATLAB as previously described [16]. Briefly, regions of interest (ROIs) were drawn on the same set of muscles on fat‐water images in MIPAV, and radial diffusivities per diffusion time were fitted with the RPBM (github.com/NYU‐DiffusionMRI/RPBM), producing membrane permeabilities and fibre diameters. Voxels with SNR < 20 [18] or fat fraction > 80% [16] were removed from ROIs prior to calculation of diffusion metrics.

### 2.5 Muscle Fat Fraction Determination

Muscle fat fraction was calculated based on fat‐water images, as previously described [33]. Muscle ROIs were drawn on five adjacent slices, of 10 mm thickness and with a 5 mm gap, centred around the thickest part of the calf. Fat fractions were calculated per muscle, per slice as the signal intensity (SI) of fat divided by the summed SI of fat and water: SI fat / (SI fat + SI water) × 100%. Mean fat fractions were then determined as the area‐weighted average of each muscle ROI across all five slices.

### 2.6 Statistical Analysis

**FIGURE 1:**
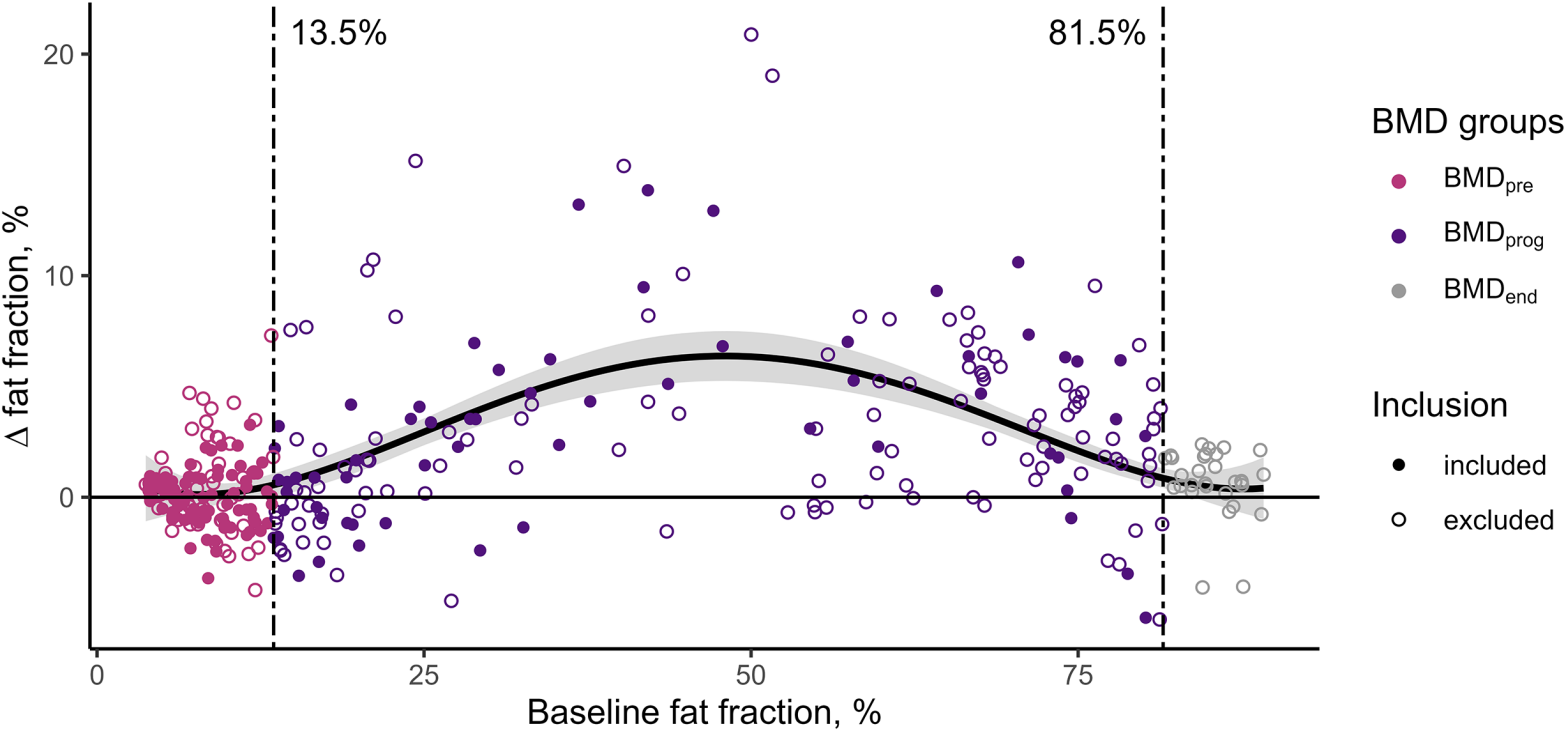
Scatterplot of fat‐fraction change over 24 months versus baseline fat fraction in patients with Becker muscular dystrophy (BMD), generated from data published in Veeger et al. [33], showing the categorisation of muscles in this study. Points show individual muscles of the upper and lower leg. The plotted line represents a sigmoidal fit to fat‐fraction change over 24 months as a function of baseline fat fraction, whereas the ribbon gives the upper and lower bounds of the fit. Thresholds for the rapid progression phase (BMD_prog_) were set as the two points where the lower bound of the fit crossed the x‐axis, which represents 0% fat‐fraction change over 24 months. Muscles were regarded as ‘preserved’ (BMD_pre_) if their baseline fat fraction was below the lower threshold (≤ 13.5%) and ‘end‐stage’ (BMD_end_) if they exceeded the upper threshold (≥ 81.5%). Closed circles show data from lower leg muscles that were also included in the current analysis. Open circles show data that were ultimately not included in this study: namely, muscles from the upper leg (thigh) or with baseline fat fractions greater than 81.5%. DOI: 10.6084/m9.figshare.28777076

All statistical analyses were performed in R (version 4.4, R Foundation for Statistical Computing, Vienna, Austria). BMD muscles were categorised into groups based on previous work in the same cohort by Veeger et al. [33], where progression of fat replacement was characterised by a sigmoidal fit to fat‐fraction change over 24 months as a function of baseline fat fraction. In this previous work, upper‐leg (thigh) scans were included in addition to the lower‐leg scans that were analysed in the current work. Based on the sigmoidal fit from Veeger et al., we identified baseline fat fraction thresholds for the rapid progression phase as the two points where the lower bound of the fit crossed the *x‐*axis (Figure 1), which represented 0% fat‐fraction change over 24 months. Muscles were regarded as follows: ‘preserved’ (BMD_pre_) if their baseline fat fraction was below the threshold at which rapid fat replacement begins (≤ 13.5%); ‘progressing’ (BMD_prog_) if they were in the range between the lower and upper thresholds (13.5% < fat fraction < 81.5%); and ‘end‐stage’ (BMD_end_) if they exceeded the upper threshold of this range (≥ 81.5%). End‐stage muscles were excluded from further analyses.

For ^31^P‐MRS metrics, a two‐way analysis of variance (ANOVA) was used to examine the main effects of group and muscle, and their interaction, at baseline. Post hoc testing was performed using Tukey's range test. For DT‐MRI‐measured membrane permeability, which was skewed and had limited data per muscle, a Kruskal–Wallis test was performed to examine the main effect of group alone. Correlations between variables at baseline were assessed via Spearman's rho.

Data from ^31^P‐MRS at baseline and 24‐month follow‐up visits were compared using a linear mixed‐effect model analysis of repeated measures with restricted maximum likelihood estimation and an autoregressive covariance structure. Random effects were modelled on a per‐patient and per‐muscle basis, including random intercepts and slopes. We determined 95% confidence intervals (CIs) and *p*‐values for the fixed effects using a Wald *t*‐distribution approximation. Post hoc analyses were performed by pairwise comparison of estimated marginal means using the Tukey method to adjust for multiple testing. Otherwise, a *p*‐value < 0.05 was considered statistically significant.

## 3 Results

### 3.1 Participant Cohort

Participants with BMD had a mean [range] age of 41.1 [18.8–66.2] years. Healthy controls had a mean [range] age of 43.0 [21.2–63.6] years and were not significantly different in age from the BMD group (Mann–Whitney U test, *p* = 0.770). Out of 23 patients with BMD, two were wheelchair‐bound and 21 were ambulant.

### 3.2 Quality Control of ^31^P‐MRS Spectra

**FIGURE 2:**
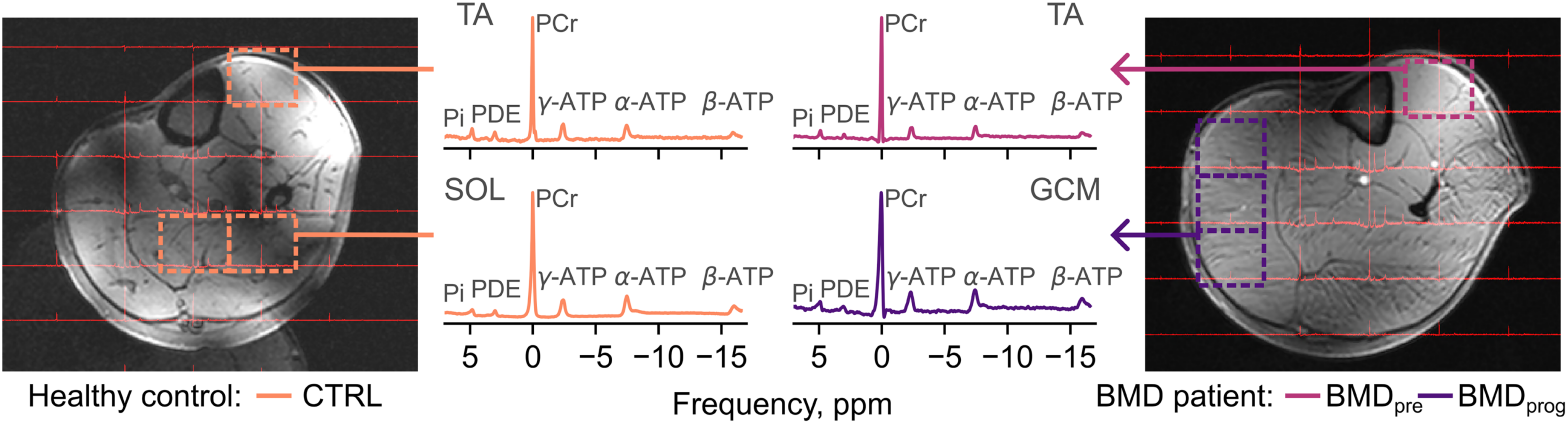
Examples of ^31^P chemical shift imaging (CSI) spectra for this study, in a control (CTRL, left) and a patient with Becker muscular dystrophy (BMD) (right). CSI grids are superimposed on T_1_‐weighted images from the left lower leg. Example spectra are shown for the tibialis anterior (TA) and soleus (SOL) muscles in the control, and the TA and gastrocnemius medialis (GCM) muscles in the BMD patient, which were in the ‘preserved’ (BMD_pre_) and ‘progressing’ (BMD_prog_) phases, respectively. Spectra from the SOL and GCM spectra were produced by alignment and averaging across multiple voxels. Ionised magnesium concentrations, [Mg^2+^], were determined via the observed shift between alpha and beta adenosine triphosphate (ATP), δ^α‐β^, and the weighted pH from inorganic phosphate (Pi), which was fitted with two peaks (Pi_a_ and Pi_b_, not shown). Also shown are spectral lines from phosphocreatine (PCr) and phosphodiesters (PDE). DOI: 10.6084/m9.figshare.28777109

**FIGURE 3:**
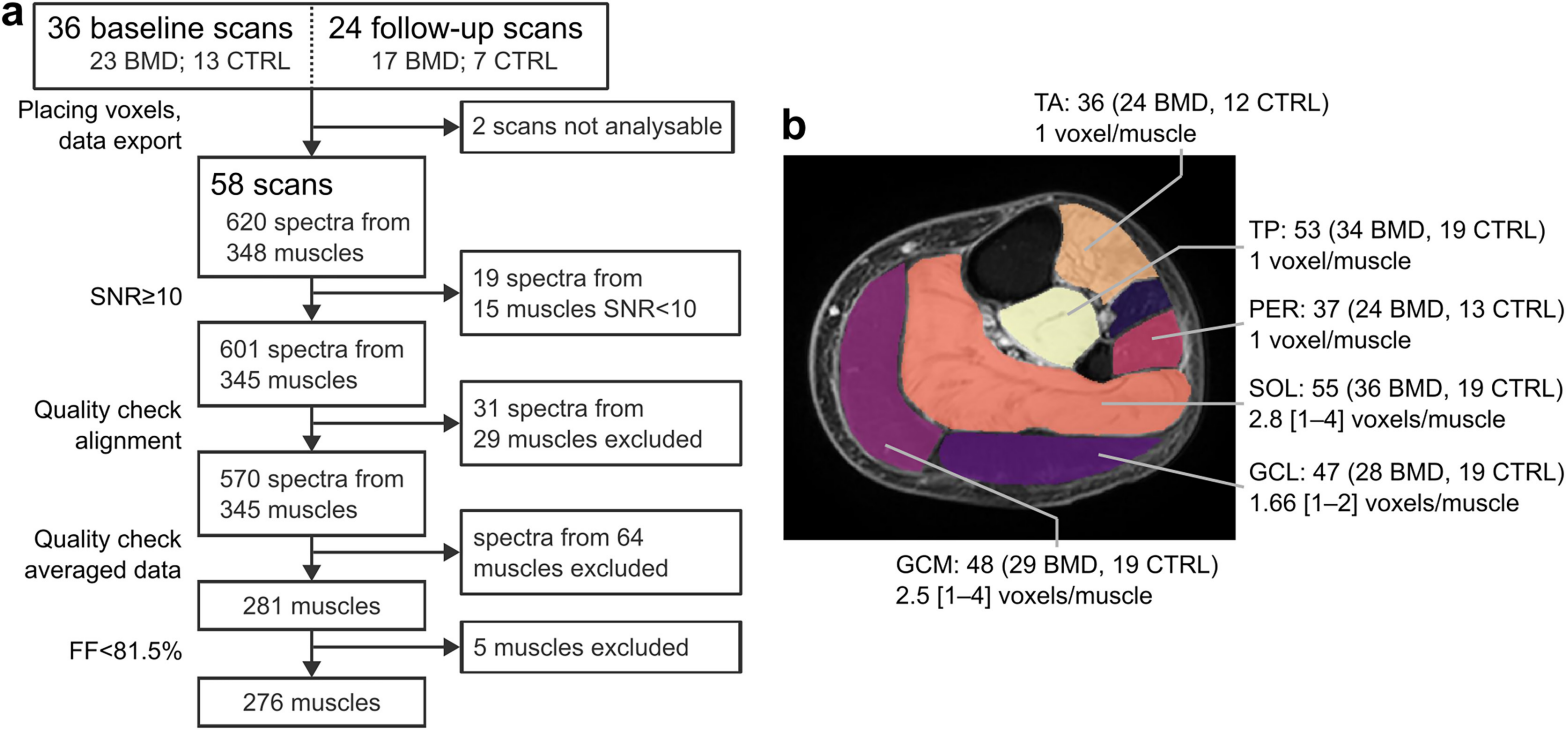
(a) Flow‐chart describing the data quality control and exclusions; and (b) a segmented chemical‐shift‐based water‐fat‐separation image of the left lower leg illustrating the number of included spectra per muscle, divided into patients with Becker muscular dystrophy (BMD) and healthy controls (CTRL). TA = tibialis anterior muscle, TP = tibialis posterior muscle, PER = peroneus muscle, SOL = soleus muscle, GCL = lateral head of the gastrocnemius, GCM = medial head of the gastrocnemius. DOI: 10.6084/m9.figshare.28777139

Representative ^31^P‐MRS spectra from a patient with BMD and healthy control are shown in Figure 2, whereas a flowchart of the quality control process, with numbers of excluded spectra, is shown in Figure 3a. Two scans, both in participants with BMD at baseline, were excluded entirely due to artefacts related to B_0_ shimming failure. Out of 620 exported spectra, 19 spectra from 15 muscles were excluded based on low SNR (< 10). In 29 muscles for which multiple spectra were exported, 31 spectra were excluded because of artefacts or alignment issues. Spectra from another 64 muscles were excluded because of artefacts after inspection of averaged spectra or spectra exported from a single voxel in a muscle (PER, TA, and TP muscles). Out of 492 fitted spectra, five (two BMD, both GCM muscle; three CTRL, one GCM, one SOL and one TA muscle) demonstrated broad PDE peak fits. These were fitted again manually in AMARES using weighting of the first 16 points and fixed zero‐ and first‐order phase terms. Finally, all five remaining muscles in the BMD_end_ category, with FF ≥ 81.5%, were also excluded. This left 276 averaged muscle spectra for statistical analysis, where the distribution of muscles is shown in Figure 3b.

### 3.3 Cross‐Sectional Comparisons

**FIGURE 4:**
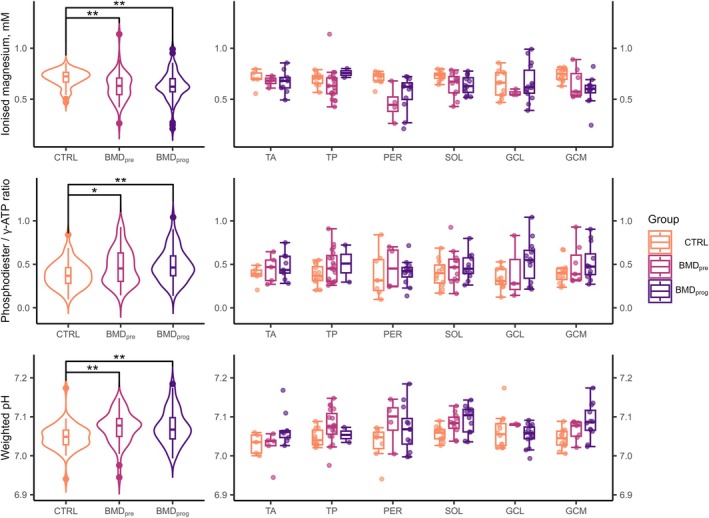
Estimated intracellular ionised magnesium ([Mg^2+^] top row), phosphodiester‐to‐γ‐adenosine‐triphosphate (PDE/γ‐ATP) ratios (middle row), and pH values (bottom row) from skeletal muscle ^31^P‐MRS. Violin and box plots (left) show results for all muscles in healthy controls (CTRL), preserved muscles in patients with Becker muscular dystrophy (BMD_pre_) and muscles in the rapidly‐progressing fat‐replacement phase (BMD_prog_). For boxplots, thick lines represent median values, hinges represent the interquartile range (IQR), and whiskers represent hinges ±1.5 × IQR. Further boxplots (right) show results for the three groups in selected muscles of the lower leg. GCL/GCM = gastrocnemius lateralis/medialis, PER = peroneus longus, SOL = soleus, TA/TP = tibialis anterior/posterior. Statistically‐significant differences are highlighted: ** = *p* < 0.05, ** = *p* < 0.01, *** = *p* < 0.001. DOI: 10.6084/m9.figshare.28777124

Figure 4 shows violin plots of [Mg^2+^], PDE/γ‐ATP, and weighted pH for all control and BMD groups at baseline, together with boxplots where results are sub‐divided by muscle. The latter show that the variability tends to be higher in BMD muscles compared to control muscles.

### 3.4 Intracellular [Mg^2+^] Was Lower in BMD Muscles Than in Healthy Controls

Two‐way ANOVA indicated that ^31^P‐MRS‐derived free [Mg^2+^] was significantly different between groups, with *F*(2, 152) = 8.3, and *p* < 0.001, but not between individual muscles: *F*(2, 152) = 1.9, *p* = 0.106. There was no significant interaction between group and muscle: *F*(2, 152) = 1.38, *p* = 0.195. Post hoc tests showed significant differences between control muscles and both BMD_pre_ (adj. *p* = 0.003) and BMD_prog_ (adj. *p* = 0.001) muscles, but not between the two BMD stages (adj. *p* = 0.997). Baseline median [IQR] Mg^2+^ concentrations were 0.72 [0.10] mM in control muscles, 0.63 [0.16] mM in BMD_pre_, and 0.62 [0.13] mM in BMD_prog_ muscles.

### 3.5 PDE/γ‐ATP Was Higher in BMD Muscles Than in Healthy Controls

PDE/γ‐ATP was also significantly different between groups, with *F*(2, 125) = 5.7, and *p* = 0.011, but again, not between individual muscles: *F*(2, 152) = 0.4, *p* = 0.846. There was no significant group × muscle interaction: *F*(2, 152) = 0.36, *p* = 0.961. Post hoc testing showed a significant difference between BMD_prog_ muscles and control muscles (adj. *p* = 0.005), and between BMD_pre_ muscles and control muscles (adj. *p* = 0.027), but not between the two BMD stages (adj. *p* = 0.903). The median [IQR] PDE/γ‐ATP at baseline was 0.37 [0.18] in control muscles, 0.45 [0.33] in preserved muscles and 0.46 [0.23] in progressing muscles.

### 3.6 Weighted pH Was Higher in BMD Muscles Than in Healthy Controls

Significant differences were also observed in weighted pH between groups, with *F*(2, 152) = 8.5, and *p* < 0.001, but not between individual muscles, with *F*(2, 152) = 1.9, and *p* = 0.083; however, there was no significant group × muscle interaction: *F*(2, 152) = 1.6, *p* = 0.101. Specifically, there were differences between control muscles and both BMD_pre_ (adj. *p* = 0.003) and BMD_prog_ (adj. *p* = 0.001) muscles, but not between the two BMD stages (adj. *p* = 0.976). Median [IQR] values at baseline were 7.05 [0.04] in control muscles, 7.08 [0.05] in BMD_pre_ muscles, and 7.07 [0.05] in BMD_prog_ muscles.

### 3.7 DT‐MRI‐Measured Membrane Permeability Did Not Differ Between Groups

RPBM‐DT‐MRI at 24‐month follow‐up showed similar permeabilities between groups, with *H*(2) = 3.4, *p* = 0.187. Markedly high‐ and low‐permeability outliers were evident in all groups (Supplementary Figure S1). Median [IQR] values were 0.046 [0.065] μm/ms in control muscles, 0.033 [0.041] μm/ms in BMD_pre_ muscles and 0.088 [0.098] μm/ms in BMD_prog_ muscles.

### 3.8 Inter‐Parameter Correlations at Baseline

Comparing metrics in the whole cohort, [Mg^2+^] was not significantly correlated with PDE/γ‐ATP (Spearman's ρ = −0.01, *p* = 0.929) but was correlated with weighted pH (ρ = −0.38, *p* ≪ 0.001). PDE/γ‐ATP was not significantly correlated with weighted pH (ρ = 0.07, *p =* 0.380). RPBM permeability did not significantly correlate with [Mg^2+^], PDE/γ‐ATP, or with weighted pH, with ρ = 0.03, *p* = 0.908; ρ = 0.19, *p* = 0.484; and ρ = −0.50, *p* = 0.051, respectively.

### 3.9 Longitudinal Comparisons

**FIGURE 5:**
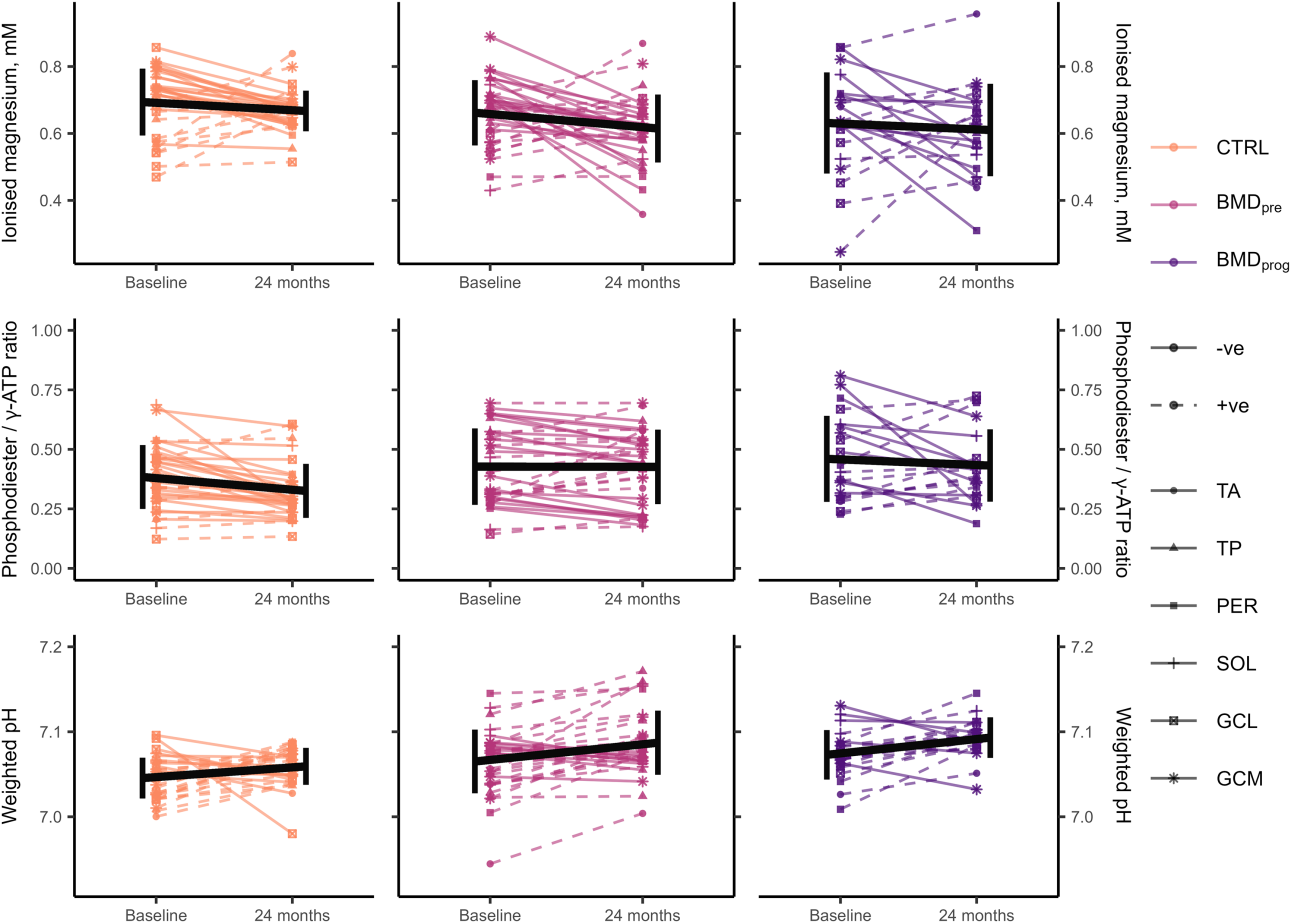
Longitudinal changes in skeletal‐muscle‐^31^P‐MRS‐derived intracellular ionised magnesium ([Mg^2+^] top row), phosphodiester‐to‐γ‐adenosine‐triphosphate (PDE/γ‐ATP) ratios (middle row) and pH values (bottom row) from baseline to 24‐month follow‐up, pooling results from individual muscles. Line plots show changes in ^31^P‐MRS metrics in healthy controls (CTRL), as well as in preserved muscles and muscles in the rapidly‐progressing fat‐replacement phase in patients with Becker muscular dystrophy (BMD_pre_ and BMD_prog_, respectively). Black lines show the overall mean (horizontal line) and the standard deviation (vertical lines) at each study visit. GCL/GCM = gastrocnemius lateralis/medialis, PER = peroneus longus, SOL = soleus, TA/TP = tibialis anterior/posterior. *p*DOI: 10.6084/m9.figshare.28777163

Figure 5 shows line plots describing the longitudinal variation of [Mg^2+^] and PDE/γ‐ATP for all control and BMD groups from baseline to 24‐month follow‐up, with individual muscles from each participant pooled together. No muscles transitioned from BMD_pre_ to BMD_prog_, whereas two BMD_prog_ muscles transitioned to end stage at follow‐up and were excluded from longitudinal analysis.

### 3.10 Intracellular [Mg^2+^] Did Not Change Longitudinally, but Group Differences Remained

The linear mixed‐effect model recapitulated our cross‐sectional findings, with group significantly predicting [Mg^2+^] status, giving *b* = 0.06, 95% CI [0.00, 0.12], t (33) = 2.04, adj. *p* = 0.004; however, visit and muscle did not significantly predict [Mg^2+^] (adj. *p* = 0.232 and 0.073, respectively), nor did any of the interactions between the three factors (adj. *p* > 0.126). Estimated marginal means (SE) per group, adjusting for visit and muscle, were 0.67 (0.01) for controls, 0.61 (0.02) for BMD_pre_ and 0.63 (0.02) for BMD_prog_, where the BMD_pre_ group was significantly different from controls (adj. *p* = 0.014), but there was no significant difference between controls and BMD_prog_ (adj. *p* = 0.150), or BMD_pre_ and BMD_prog_ (adj. *p* = 0.711).

### 3.11 No Longitudinal Changes or Group or Muscle Differences Were Observed for PDE/γ‐ATP

For the PDE/γ‐ATP model, none of the three factors—group, visit and muscle—significantly predicted PDE/γ‐ATP (adj. *p* = 0.159, 0.329 and 0.758, respectively), and there were no significant interactions between these (adj. *p* > 0.220).

### 3.12 Weighted pH Did Not Change Longitudinally, but Showed Differences Between Muscles

The weighted pH linear mixed‐effect model showed muscle significantly predicted pH, with *b* = 0.01, 95% CI [−0.01, 0.03], t(139) = 1.09 and adj. *p* = 0.006, while visit or group alone were not significant (adj. *p* = 0.053 and 0.064), and there were no significant interactions (adj. *p* > 0.056). Looking at estimated marginal means (SE) per muscle, pH was seen to be higher in the PER muscle as compared to the TA, with 7.08 (0.01) versus 7.06 (0.01), (*p* = 0.044); the SOL muscle as compared to the TA, with 7.08 (0.01) versus 7.06 (0.01), (*p* = 0.002); and in the SOL muscle as compared to the GCL, with 7.08 (0.01) versus 7.07 (0.01).

## 4 Discussion

In this study, we investigated several candidate MR markers reflecting skeletal muscle membrane permeability as possible biomarkers for disease activity in patients with BMD. To this end, we separated muscles from patients with BMD into two groups, based on their expected rate of fat replacement over time: ‘preserved’ muscles, and muscles undergoing rapid progression. We showed that [Mg^2+^] was decreased in both groups of muscles as compared to healthy controls, whereas weighted pH and PDE/γ‐ATP were increased in these muscles. Weighted pH and [Mg^2+^] were also significantly correlated with one another. No differences were found in membrane permeability measures obtained using the RPBM between BMD muscles and controls, and these were also not correlated with ^31^P‐MRS metrics. Longitudinal analysis revealed no relation between study visit alone and the MR metrics. The mixed‐effect model showed weighted pH was significantly higher, thus more alkaline, in the SOL muscle as compared to the TA, the PER muscle as compared to the TA, and in the SOL muscle as compared to the GCL. Furthermore, group predicted [Mg^2+^] status; both BMD_pre_ and BMD_prog_ were significantly different from controls, but not from each other, in line with the cross‐sectional analysis at baseline.

### 4.1 Magnesium as a Biomarker for Disease Activity in BMD

Reduction in intracellular ionised magnesium is hypothesised to reflect increased membrane permeability [10]. We showed reduced concentrations of Mg^2+^ in BMD muscles as compared to controls, even in preserved muscles, which may imply membrane permeability is increased in all muscles in patients with BMD independent of the disease stage. Our finding is in agreement with previous work in patients with DMD [10]. In that study, estimated Mg^2+^ concentrations tended to be lower than those seen here, with average values of mean (SD) = 0.53 (0.05) mM in controls and as low as 0.44 (0.06) in patients with DMD, whereas we observed median [IQR] Mg^2+^ concentrations of 0.72 [0.10] mM in controls and 0.62 [0.13] mM at minimum in patients with BMD. These differences are not likely to be due to the influence of pH, as our estimated pH values tended to be lower than those reported by Reyngoudt and colleagues, which would lead to higher apparent Mg^2+^ concentrations. Instead, they could be explained by differences between paediatric and adult cohorts, or by methodological differences: 7 T versus 3 T, or CSI versus pulse‐acquire acquisitions. Children have a lower upper limit of normal for serum Mg levels [34], and previous work by Niermann et al. has shown that [Mg^2+^] tends to be lower in juvenile dermatomyositis patients and controls versus adult dermatomyositis patients and controls [35]. Regarding technical differences, previous work in an adult cohort by members of our group, using a similar approach to Reyngoudt and colleagues, showed higher median [IQR] Mg^2+^ concentrations of 0.64 [0.12] mM in younger adults, between 24 and 40 years of age, and 0.53 [0.11] mM in older adults, over the age of 80 years [24]. Franke et al. [36], on the other hand, performed CSI experiments in young adult volunteers at 7 T using a different approach for calculating [Mg^2+^] [37, 38]. By back‐calculating $\delta_{\mathrm{obs}}^{\alpha -\beta }$ values from their work, and combining these with their reported pH values in Equation (3), we were able to derive [Mg^2+^] values of a similar magnitude to those of our healthy controls, ranging from 0.55 to 0.70 mM. These results suggest that differences between adult and paediatric cohorts explain some of the differences in [Mg^2+^] values reported here as compared to the literature, with methodological differences perhaps having a smaller effect.

We did not observe inter‐muscle differences in [Mg^2+^] in any of the groups or sub‐groups studied. Reyngoudt and colleagues also observed no differences between the muscles of the forearm and the anterior and posterior compartments of the lower leg in DMD [10]; however, Franke et al. showed small but clear differences between lower‐leg muscles in a small group of healthy controls using a high‐resolution 3D CSI acquisition [36]. The lack of inter‐muscle differences here could be attributed to our lower‐resolution 2D CSI acquisition; our larger voxels likely included more signal from surrounding muscles, limiting our sensitivity to differences. Overcoming this limitation with high‐resolution [Mg^2+^] mapping in our patient cohort would be of great interest for understanding muscle involvement in BMD.

Our linear mixed‐effect model analysis showed no relationships between study visit and [Mg^2+^] status. This may indicate that [Mg^2+^] had already decreased to a stable level in BMD muscles. Indeed, earlier work in patients with DMD showed no differences at one‐year follow‐up, with the authors positing that alterations in [Mg^2+^] either progress slowly after an initial change, or are stable, showing continuous disease activity [10]. It is not clear to what extent intracellular ionised magnesium can be influenced by factors like diet, exercise or stress, and whether it has a circadian rhythm, as has been shown for plasma ionised magnesium [39]. Future work should investigate the same‐day repeatability of ^31^P‐MRS assays of [Mg^2+^] to evaluate their usefulness for monitoring disease activity.

### 4.2 Phosphodiesters Are Elevated in BMD

The PDE resonance observed in skeletal muscle ^31^P spectra primarily arises from the phospholipid glycerol 3‐phosphorylcholine [40], which is essential to the function and integrity of the cell membrane. We showed statistically‐significant differences in PDE/γ‐ATP between patients with BMD and healthy control muscles, but did not observe differences between preserved muscles and progressing muscles. Previous work by our group in the same cohort [18], and in a different cohort [17], showed similar results, with increased PDE/γ‐ATP in patients with BMD in some fat‐replaced muscles and one non‐fat‐replaced muscle. Further previous work by our group, and by others, has also shown elevated PDE/γ‐ATP in patients with DMD versus controls [10, 41]. Unlike these studies, we divided patients into muscles likely to be preserved and muscles likely to progress based on baseline fat fraction, showing elevation of phosphodiesters is not disease phase specific. Non‐fat‐replaced muscles in our previous work were defined more conservatively than the BMD_pre_ group here, being based on the range of fat fractions seen in healthy control muscles [18]. Notably, neither the current study nor our previous studies observed any significant inter‐muscle differences in PDE levels in controls or in patients with BMD, though Reyngoudt and colleagues have observed such differences in DMD [10]. One study evaluated the effect of treatment on ^31^P‐MRS parameters in DMD and showed PDE/ATP was significantly lower in DMD boys who received micro‐dystrophin gene therapy compared to untreated DMD boys. The PDE/ATP in treated DMD boys was higher compared to controls, reflecting partial normalisation [42]. This strengthens its potential use as a biomarker reflecting disease progression.

Considering our longitudinal analyses, we saw no effect of visit or group on PDE/γ‐ATP, consistent with previous work in DMD, where concentrations remained elevated, but stable, at 12‐month and 24‐month follow‐up [43]. Again, diurnal variation should be considered for future studies, as a ^1^H‐MRS study has demonstrated a 10% increase in total choline concentrations in the human brain between 07:00 and 12:00 [44], where the total choline resonance contains contributions from glycerophosphorylcholine.

### 4.3 Skeletal Muscle Weighted pH Elevated in BMD

Elevated skeletal muscle pH in DMD has been proposed as an outcome of a chain of events beginning with membrane leakiness and leading to disrupted ionic homeostasis [9]. A small ^31^P‐MRS study in DMD showed pH in boys who had received micro‐dystrophin gene therapy was similar to that in controls, whereas in untreated DMD boys, pH was significantly elevated. Here, we show significantly elevated pH values in both preserved and progressing BMD muscles as compared to healthy controls. Reyngoudt and colleagues also showed two subgroups of patients with DMD with reduced [Mg^2+^]: one with more‐alkaline ^1^H‐MRS‐derived intracellular pH, similar to our patient group, and another where pH was unchanged [10]. Weighted pH also showed a negative correlation with [Mg^2+^], in agreement with Reyngoudt and colleagues [10]. Notably, our previous study in this cohort, which used a single inorganic phosphate peak for ^31^P‐MRS pH determination as opposed to those that used the two‐peak weighted pH used here [8], showed no significant changes in pH in BMD. However, we have previously shown pH differences in another BMD cohort using the single‐peak method, as have others [45, 46].

Longitudinally, we observed that muscle significantly predicted weighted pH. Indeed, pH was seen to be significantly higher in the PER and SOL muscles, as compared to the TA, for example. Such inter‐muscle differences in weighted pH have been observed before: Reyngoudt and colleagues showed differences between the muscles of the forearm and the lower leg in DMD patients, but not in controls [10].

### 4.4 DT‐MRI Membrane Permeability Did Not Differ Between Groups or Correlate With 31P‐MRS

We observed no group differences in RPBM‐derived membrane permeabilities, similar to earlier work in the same cohort using DT‐MRI mean diffusivity [18]—a less‐specific permeability marker. Further, no correlations were observed with PDE/γ‐ATP or [Mg^2+^], perhaps because these metrics reflect different aspects of membrane integrity, or because fibrosis is not explicitly modelled in the RPBM. Unpublished work by our group (data not shown) suggests that inclusion of an additional long diffusion time, of 530 ms, gives RPBM permeability values that are more in line with Tanner's early ex vivo experiments [11], and may be more comparable with the ^31^P‐MRS metrics described here.

## 5 Limitations

The stringent quality control required for accurate Mg^2+^ and pH estimation in this study led to some exclusions, and thus reduced statistical power. Accurate determination of [Mg^2+^] requires that the α‐ and β‐ATP peaks in the upfield part of the ^31^P spectrum are adequately resolvable, and that pH can be reliably estimated from the downfield Pi peaks. Thus, good‐quality B_0_ shimming and adequate SNR are essential. Muscle fat fraction was calculated as an area‐weighted average across five slices, covering a 7‐cm thick slab, whereas ^31^P‐MRS voxels were non‐localised in the slice direction, covering a 12‐cm‐thick volume. It is known that the fat distribution in DMD and BMD muscles is non‐linear [47, 48], so fat fractions estimated from whole muscles may have been marginally higher or lower than the values we show here. However, we expect the influence on our results to be limited, as we used fat fractions to categorise muscles as progressing and preserved, and did not numerically compare them with MRS metrics. A software update took place on the 7 T, roughly between the baseline scans and the follow‐up scans. However, this update is not expected to have had an effect on the metrics studied here. Follow‐up scans were not always performed around the same time of day as the baseline scans. At baseline, most participants were scanned in the late afternoon and early evening, between around 16:30 and 18:00, whereas at 24‐month follow‐up, most participants were scanned in the late morning and early afternoon, typically between 11:00 and 13:00. In light of possible diurnal variation, this could have influenced the results, especially the longitudinal analysis and the observed outliers in those data. Further, our study did not include ^1^H‐MRS measures of intracellular pH, thus we cannot entirely discount a possible bias in our [Mg^2+^] estimates relating to the influence of extracellular contributions to our ^31^P‐MRS‐measured pH. However, Reyngoudt and colleagues have shown that [Mg^2+^] differences between DMD patients and controls persist when accounting for the influence of extracellular pH [10], and we expect similar changes here.

## 6 Future Applications of This Work

Of the metrics studied here, [Mg^2+^] and weighted pH represent the most promising biomarkers for disease activity. Both are significantly different between healthy controls and participants with BMD, but do not discriminate between different disease stages, perhaps reflecting continuous disease activity. For these to be validated as response biomarkers, they must be seen to normalise after initiation of treatment. Although there is currently no approved treatment to replicate these findings in BMD, ^31^P‐MRS could be incorporated into upcoming therapeutic trials. If a treatment effect is established in the trial, response of [Mg^2+^] and weighted pH can then be immediately investigated, obviating the need to initiate another trial for biomarker validation.

Regarding the other measures studied here, PDE/γ‐ATP may also represent a good marker for disease activity. However, the lack of group differences in our longitudinal analyses suggests it may be less sensitive than [Mg^2+^] and weighted pH. The RBPM did not show differences between patients with BMD and controls; however, promising initial results with longer diffusion times, and fibre‐size differences previously shown with the technique [16], suggest avenues for further research.

Additionally, experiments on the diurnal stability and repeatability of Mg^2+^ and PDE levels, as well as the effects of dietary intake of magnesium and recent sport activity, will contribute to a better understanding of the behaviour of these biomarkers, and are essential for implementation.

## 7 Conclusion

We show reduced intramuscular [Mg^2+^] and increased PDE/γ‐ATP and weighted pH in patients with BMD versus healthy controls, whereas DT‐MRI‐measured permeability showed no differences. Altered [Mg^2+^], PDE/γ‐ATP and weighted pH were observed throughout the different stages of the disease course, in muscles in both the preserved and progressing phases. Inclusion of ^31^P‐MRS in therapeutic trials could be useful to further explore and establish [Mg^2+^], PDE/γ‐ATP and weighted pH as response biomarkers. We found no robust alterations of these markers at follow‐up, suggesting the markers reflect a continuous disease activity.

## Author Contributions

Conceptualisation: H.E.K., D.C. Data curation: M.T.H., N.M.V. Formal analysis: E.J.S., D.C. Data interpretation: E.J.S., E.H.N., H.E.K, D.C. Writing and editing: E.J.S., M.T.H., N.M.V., E.H.N., H.E.K., D.C.

## Conflicts of Interest

H.E. Kan reports research support from Philips Healthcare during the conduct of the study, and trial support from ImagingDMD‐UF outside the submitted work; all reimbursements for H.E. Kan were received by the LUMC, and no personal financial benefits were received. E.H. Niks discloses ad hoc consultancies for BioMarin, Entrada Therapeutics, Edgewise, Italfarmaco, Pfizer, Roche, Sarepta Therapeuticals and Solid Bioscience, (all reimbursements were received by the LUMC; no personal financial benefits were received); reports receiving grants from Duchenne Parent Project, Prinses Beatrix Spierfonds, the European Union, Dutch Research Council, Spieren voor Spieren and Pfizer; and has been site principal investigator for clinical trials conducted by BioMarin, Edgewise, Fibrogen, Italfarmaco, ML Bio, NS Pharma, Reveragen, Santhera Pharmaceuticals, Sarepta, Alexion, Janssen and Argnx outside the submitted work. All other authors report no disclosures.

## Supporting information


**Figure S1:** Estimated membrane permeabilities, κ, derived from the random permeable barrier model (RPBM) as applied to diffusion‐tensor MRI data acquired in skeletal muscle. Violin and box plots (top left) show values for κ for all muscles in healthy controls (CTRL), preserved muscles in patients with Becker muscular dystrophy (BMD_pre_) and muscles in the rapidly‐progressing fat‐replacement phase (BMD_prog_). For boxplots, thick lines represent median values, hinges represent the interquartile range (IQR), and whiskers represent hinges ±1.5 × IQR. Further boxplots (top right) show results for the three groups in selected muscles of the lower leg. Finally, scatter plots (bottom row) compare κ values to ionised magnesium [Mg^2+^], phosphodiester‐to‐γ‐adenosine‐triphosphate (PDE/γ‐ATP) ratios and pH values from ^31^P‐MRS, respectively. Here, black lines show a linear regression fit to the data, and grey bands represent the 95% confidence intervals of the fit. No statistically significant correlations were observed between κ and ^31^P‐MRS metrics. GCL/GCM = gastrocnemius lateralis/medialis, PER = peroneus longus, SOL = soleus, TA/TP = tibialis anterior/posterior. DOI: 10.6084/m9.figshare.30032392.

## Data Availability

The data that support the findings of this study are available from the corresponding authors upon reasonable request.
