# Supplementary material for: Skeletal Muscle Membrane Permeability Markers Derived From 31P‐MRS May Reflect Disease Activity in Becker Muscular Dystrophy
Source: NMR Biomed. 2025 Oct 7;38(11):e70155. doi: 10.1002/nbm.70155 (PMC12504916; doi:10.1002/nbm.70155)
Supplement: Supplementary file 1 — Figure S1: Estimated membrane permeabilities, κ, derived from the random permeable barrier model (RPBM) as applied to diffusion‐tensor MRI data acquired in skeletal muscle. Violin and box plots (top left) show values for κ for all muscles in healthy controls (CTRL), preserved muscles in patients with Becker muscular dystrophy (BMDpre) and muscles in the rapidly‐progressing fat‐replacement phase (BMDprog). For boxplots, thick lines represent median values, hinges represent the interquartile range (IQR), and whiskers represent hinges ±1.5 × IQR. Further boxplots (top right) show results for the three groups in selected muscles of the lower leg. Finally, scatter plots (bottom row) compare κ values to ionised magnesium [Mg2+], phosphodiester‐to‐γ‐adenosine‐triphosphate (PDE/γ‐ATP) ratios and pH values from 31P‐MRS, respectively. Here, black lines show a linear regression fit to the data, and grey bands represent the 95% confidence intervals of the fit. No statistically significant correlations were observed between κ and 31P‐MRS metrics. GCL/GCM = gastrocnemius lateralis/medialis, PER = peroneus longus, SOL = soleus, TA/TP = tibialis anterior/posterior. DOI: 10.6084/m9.figshare.30032392. [file NBM-38-e70155-s001.docx]

**Skeletal muscle membrane permeability markers derived from ^31^P-MRS may reflect disease activity in Becker muscular dystrophy**

**N.M.R. IN BIOMEDICINE**

Esther J. Schrama^1,3^, Melissa T. Hooijmans^2^, Nienke M.van de Velde^1,3^, Erik H. Niks^1,3^, Hermien E. Kan^3,4^*, Donnie Cameron^4,5^*

1. Department of Neurology, Leiden University Medical Center, Leiden, NL;

2. Department of Human Movement Sciences, Faculty of behavioural and movement sciences, VU University, Amsterdam, NL;

3. Duchenne Center Netherlands;

4. C.J. Gorter MRI Center, Department of Radiology, Leiden University Medical Center, Leiden, NL;

5. Department of Medical Imaging, Radboud University Center, Nijmegen, NL

***Correspondence:** Donnie Cameron, Department of Medical Imaging (Route 766), Radboud University Medical Center, Geert Grooteplein 10, 6525 GA Nijmegen, The Netherlands. Tel.: +31 24 361 8908; E-mail: [Donnie.Cameron@radboudumc.nl](mailto:Donnie.Cameron@radboudumc.nl)

Hermien E. Kan, C.J. Gorter MRI Center, Department of Radiology-C3Q, Leiden University Medical Center, Albinusdreef 2, 2333 ZA Leiden, The Netherlands. Tel.: +31 71 526 6097; E-mail: [H.E.Kan@lumc.nl](mailto:H.E.Kan@lumc.nl)

**SUPPLEMENTARY FIGURE S1**


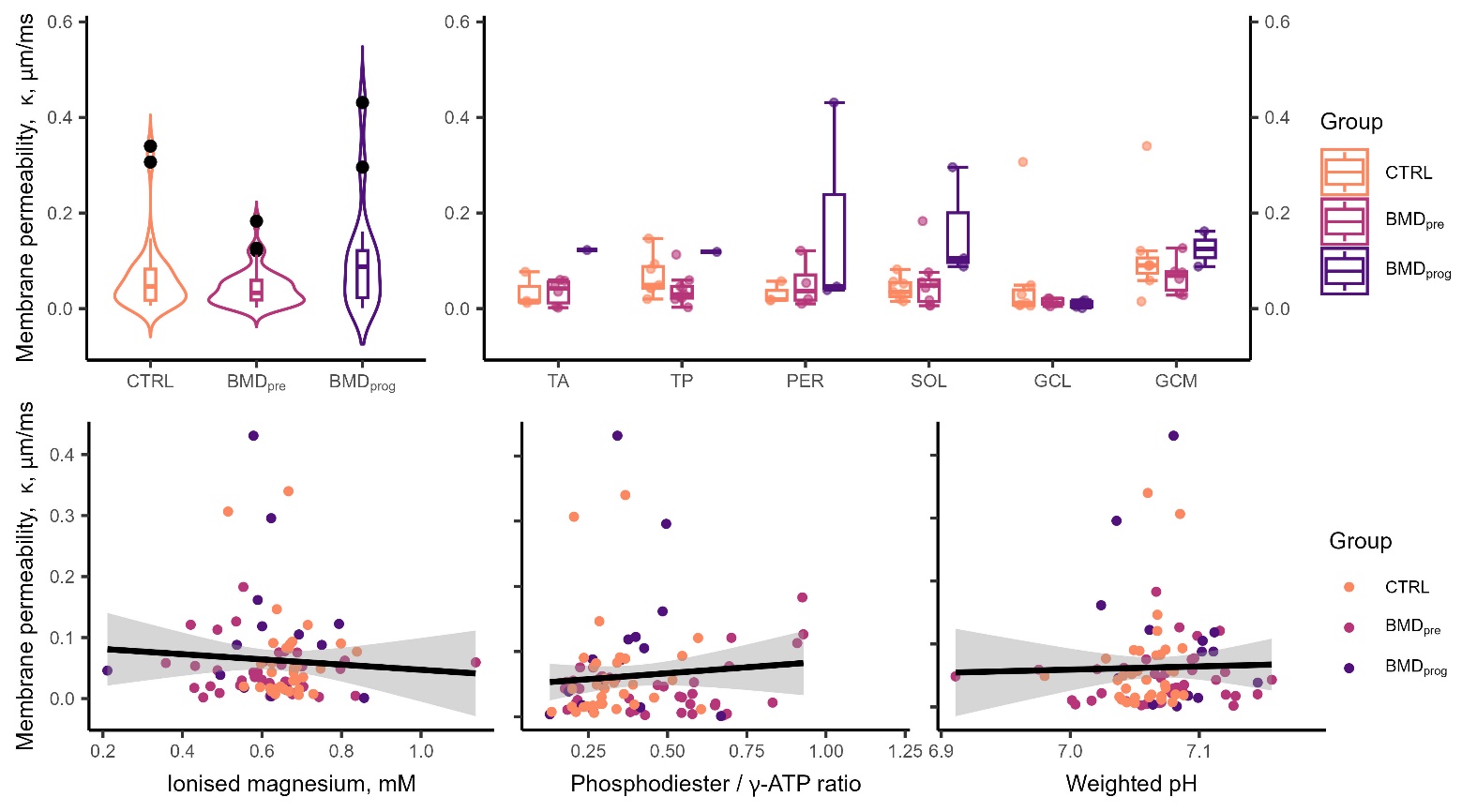


**Supplementary Figure S1.** *Estimated membrane permeabilities, κ, derived from the random permeable barrier model (RPBM) as applied to diffusion-tensor MRI data acquired in skeletal muscle. Violin and box plots (top left) show values for κ for all muscles in healthy controls (CTRL), preserved muscles in Becker muscular dystrophy patients (BMD_pre_), and muscles in the rapidly-progressing fat-replacement phase (BMD_prog_). For boxplots, thick lines represent median values, hinges represent the interquartile range (IQR), and whiskers represent hinges ± 1.5 × IQR. Further boxplots (top right), show results for the three groups in selected muscles of the lower leg. Finally, scatter plots (bottom row) compare κ values to ionised magnesium [Mg^2+^], phosphodiester-to-γ-adenosine-triphosphate (PDE/γ-ATP) ratios, and pH values from ^31^P-MRS, respectively. Here, black lines show a linear regression fit to the data, and grey bands represent the 95% confidence intervals of the fit. No statistically-significant correlations were observed between κ and ^31^P-MRS metrics. GCL/GCM = gastrocnemius lateralis/medialis, PER = peroneus longus, SOL = soleus, TA/TP = tibialis anterior/posterior. DOI: 10.6084/m9.figshare.30032392*
